# Progress in Endoscopic and Interventional Treatment of Esophagogastric Variceal Bleeding

**DOI:** 10.1155/2022/2940578

**Published:** 2022-05-06

**Authors:** Bin Liu, Gang Li

**Affiliations:** Department of Vascular Surgery, The Second Affiliated Hospital of Shandong First Medical University, Taian 271000, China

## Abstract

Esophagogastric variceal bleeding (EGVB) is one of the main complications of portal hypertension, especially in patients with liver cirrhosis, and has a very high fatality rate. At present, the treatment methods for rupture and bleeding of gastroesophageal varices (GV) include drug therapy, compression hemostasis with three-lumen and two-balloon tube, endoscopic therapy, and interventional and surgical operations. Endoscopy and intervention or their combined application is the mainstream treatment modes in clinical practice, especially their combined application has been increasingly recognized by front-line clinicians. This article intends to discuss the application characteristics of the two treatment methods.

## 1. Gastroscopy

With the development of endoscopic treatment technology, active intervention through endoscopic gastric fundus, esophageal variceal ligation, sclerosing agent injection, and other methods has greatly improved the therapeutic effect of venous rupture and bleeding compared with the single use of drugs in internal medicine. Endoscopic sclerotherapy (EIS) is a method of treating gastric fundus esophageal varices through endoscopic injection of lauryl alcohol (polyoxyethylene lauryl ether, hereinafter referred to as sclerosing agent) to achieve hemostasis and prevent rebleeding. The mechanism is to inject sclerosant into the varicose veins to cause aseptic inflammation of the varicose veins, resulting in varicose vein contracture, thrombosis, fibrous scarring, and eventually occlusion. The Japanese clinical guidelines for liver cirrhosis [1, 2] recommend EIS as the first choice for primary prevention of esophageal variceal bleeding.

Endoscopic ligation therapy (EVL) is to ligate the lower esophageal varices to occlude the veins, so as to relieve the varices and reduce the risk of rebleeding. EVL needs to be performed in several times, every 2 to 4 weeks, until the varicose veins are eliminated, so as to achieve the purpose of hemostasis and prevention of rebleeding. However, in the postoperative period of necrosis and scab removal, there is a risk of massive bleeding, and complications such as pain, esophageal ulceration, and perforation may occur.

Both EVL and EIS directly treat submucosal varicose veins under endoscope to effectively control EVB in patients with liver cirrhosis [3, 4]. Endoscopic treatment is currently the main recommended treatment for esophageal varices in national guidelines [5]. For acute bleeding, the hemostasis rate of EVL and EIS can reach 90% to 95% and can effectively reduce the rate of rebleeding [6]. The biggest advantage of gastroscopy is that it can be repeatedly treated for the disease. Multiple treatments under a gastroscope can gradually reduce varices or disappear vascular occlusion, so as to achieve the purpose of treating esophagogastric variceal bleeding [7]. Compared with conventional surgical methods, EVL and EIS have obvious advantages: wide indications, convenient operation, high success rate of hemostasis, and fewer complications. However, the persistent existence of portal hypertension in patients with liver cirrhosis can lead to the recurrence of varicose veins and affect the long-term treatment effect. Regular observation and repeated band ligation are required. In the process of clinical practice, when the patient has massive bleeding and hemodynamic instability, the application of endoscopy has a greater risk, and endoscopic therapy is not recommended as the first choice [8, 9]. For these patients, the bleeding time is one of the important factors affecting their prognosis. Patients with less bleeding and shorter bleeding time theoretically predict a better prognosis. However, during the time period from the patient's admission to the emergency department of the hospital until the definitive treatment, emergency intervention schemes can be taken, such as three-lumen two-balloon catheter compression hemostasis or interventional therapy under the emergency green channel.

## 2. Interventional Therapy

There are many treatment methods for variceal bleeding, which are described in detail in the guidelines [10]. Among them, interventional therapy is a subject that has developed rapidly in recent years. Because of its minimal invasiveness and effectiveness, it is widely used in clinical practice, mainly including partial splenic artery embolization (PSE), percutaneous transhepatic coronary venous embolization (percutaneous transhepatic coronary embolization), variceal embolization (PTVE), balloon-occluded retrograde transvenous obliteration (BRTO), portal stenting, and transjugular intrahepatic portosystemic shunt (TIPS). Interventional therapy also has the advantages of less trauma, short preoperative preparation time, quick hemostasis, and wide indications.

### 2.1. Percutaneous Transhepatic and Gastric Coronary Venous Embolization

Percutaneous transhepatic and gastric coronary vein embolization (PTVE) refers to percutaneous puncture of intrahepatic portal vein branches under the guidance of ultrasound and then superselected into varicose vein branches such as gastric coronary vein/short gastric vein and injected with embolic materials to block bleeding, esophagus, gastric varices, to achieve the purpose of treatment and prevention of variceal bleeding. Commonly used embolic materials include hardeners (polyurethane), gelatin sponges, and spring steel rings. As a minimally invasive treatment method, it has achieved good therapeutic effect and low complication rate [11], and the treatment effect is better if combined with partial splenic artery embolization [12]. Due to the puncture complications of percutaneous transhepatic and gastric coronary venous embolization and the possibility of increasing portal pressure and causing or aggravating ascites, the application of percutaneous transhepatic and gastric coronary venous embolization has been reduced for a period of time. In recent years, endoscopic treatment of massive gastric variceal bleeding has a high failure rate in clinical practice [13], which makes percutaneous transhepatic and gastric coronary vein embolization receive due attention. Therefore, in view of the unique advantages of PTVE in the treatment of gastric fundus varices [14], percutaneous transhepatic and gastric coronary vein embolization is still worthy of active exploration for patients with portal hypertension, especially for bleeding patients who have failed endoscopic therapy.

### 2.2. BRTO for Varicose Veins

BRTO refers to inserting a balloon catheter into the outflow end of the gastrorenal or gastric shunt via the femoral vein or internal jugular vein, inflating the balloon to stop blood flow, and injecting a sclerosing agent to eliminate varicose veins [15]. As early as 1984, Olson et al. [16] tried balloon occlusion of the gastrorenal outflow tract and successfully completed the first case of GV sclerotherapy using ethanol. In 2003, a small randomized controlled study in South Korea compared BRTO and TIPS in the treatment of 14 patients with active GV bleeding and gastrorenal shunt, and the results showed that there was no significant difference in immediate hemostasis, rebleeding, and hepatic encephalopathy between the two groups [17]. Hong et al. compared the efficacy of BRTO and endoscopic tissue glue injection in the treatment of acute GV bleeding, and the results showed that there was no significant difference in the success rate of hemostasis, complications, and mortality between the endoscopic treatment group and the BRTO group, while the rebleeding rate in the treatment group was not significantly different higher than the BRTO group (71% vs. 15%) [18]. A large retrospective study evaluating the clinical outcomes of 213 patients in 6 university hospitals who received BRTO for gastric variceal bleeding found that BRTO had a technical success rate of 97% and was associated with surgery-related complications. The incidence of pulmonary embolism and renal infarction was 4.4%, the rate of GV disappearance was 52.3%, and the rate of marked remission was 2.8% [19]. After a mean follow-up of 3 years, 39 patients (18.3%) had rebleeding, 7 with GV bleeding, 18 with EV bleeding, 4 with nonvariceal bleeding, and 10 with unknown cause [19]. Cho et al. [20] analyzed 49 patients with GV complicated by spontaneous shunts. The success rate of BRTO was 84%, 2 died of perioperative complications, and there was no variceal recurrence or rebleeding. The clinical effect was significant. BRTO has more clinical application experience in Japan and South Korea and has been published in most papers in the world. The number of cases in Europe and the United States has also gradually increased in recent years. However, the current domestic development of BRTO technology is not satisfactory. On the one hand, some scholars do not pay enough attention to it, and they still unilaterally pursue reducing pressure in terms of concept. On the other hand, there is a lack of suitable interventional devices.

### 2.3. Partial Splenic Artery Embolization

Partial splenic embolization (PSE) is to infarct part of the spleen by embolizing the internal splenic artery and reduce the damage of splenic parenchyma to peripheral blood cells and is mainly used to relieve clinical symptoms of hypersplenism [21]. At the same time, it reduces portal venous pressure due to decreased splenic artery blood flow [22]. Clinically, it is combined with esophagogastric vein ligation for the control and prevention of EVB [23–26]. Clinically, partial splenic artery embolization is mainly used in patients with hypersplenism. This technique can cause partial splenic infarction by embolizing about 50% to 60% of the spleen area, reducing the destructive effect of spleen parenchyma on peripheral blood cells and increasing peripheral blood white blood cell, blood cell, and platelet counts, thereby improving hypersplenism [27]. However, whether partial splenic artery embolization can reduce portal pressure is still controversial, so there are few reports on the prevention of variceal bleeding in cirrhotic portal hypertension by partial splenic artery embolization alone [28]. In recent years, reports of partial splenic artery embolization combined with other treatments for the treatment and prevention of variceal bleeding have increased [29, 30], which is equivalent to minimally invasive splenectomy plus devascularization and achieved a certain therapeutic effect. It has also been reported that partial splenic artery embolization combined with variceal ligation can reduce the incidence of rebleeding and the mortality of patients [24]. However, due to the acute infarction of part of the spleen after PSE, the incidence of postoperative complications is high and the severity is relatively serious, such as peritonitis, splenic abscess, pneumonia, pleural effusion, and portal vein thrombosis [31–36]. These problems limit the use of PSE in cirrhotic EVB patients, especially in patients with Child-Pugh C cirrhosis. Wang et al. [37] showed that splenic artery trunk embolization (SATE) can significantly reduce portal blood flow and increase hepatic artery blood supply in patients with portal hypertension; in patients with hypersplenism and liver function status after SATE, the degree of esophageal and gastric varices was improved.

### 2.4. TIPS for Gastric Varices

TIPS is to establish a shunt channel in the liver parenchyma between the hepatic vein and the portal vein through the internal jugular vein so that the blood flow of the portal venous system can be directly returned to the systemic circulation through the shunt channel, thereby reducing the portal pressure. Complications due to treatment of portal hypertension are reported [38]. Early studies using bare stent TIPS showed that the vast majority of GV bleeding can be controlled after successful TIPS surgery [39, 40].

The following studies have compared the clinical efficacy of TIPS and endoscopic tissue glue injection in the treatment of GV bleeding. One retrospective study found that endoscopic injection of tissue glue for GV had a higher rate of rebleeding than TIPS (30% vs. 15%). In addition, the TIPS group had longer hospital stays and higher treatment costs, suggesting that endoscopic therapy may be more cost-effective [41]. The results of another retrospective study showed that the rebleeding and mortality rates were comparable between the endoscopic treatment group and the TIPS group, but the TIPS group had a significantly higher incidence of hepatic encephalopathy than the endoscopic treatment group [42]. Lo et al. conducted a prospective randomized controlled trial of 72 patients admitted to hospital with acute GV bleeding from cirrhosis who received endoscopic tissue glue injections to control active bleeding prior to randomization [43]. Then, both TIPS and endoscopic tissue glue injection were used to prevent rebleeding of gastric fundus varices. The results showed that the GV rebleeding rate in the TIPS group was significantly lower (11% vs. 38%), but the incidence of hepatic encephalopathy in the TIPS group was higher than that in the TIPS group. High (26% vs. 3%) complication rates and survival rates were not significantly different between the two groups. A newer nonrandomized controlled study compared the effect of TIPS and endoscopic tissue glue injection in the treatment of GV bleeding, and the results showed that there was no significant difference in the control of acute bleeding, prevention of rebleeding, and survival between the two groups, but the TIPS group was postoperative. The incidence of hepatic encephalopathy is higher [43]. The above studies show that although TIPS can well control and prevent GV bleeding, its clinical efficacy is roughly equivalent to EV, and it also has a high risk of postoperative hepatic encephalopathy. In recent years, due to the application of stent graft and the control of shunt diameter, the clinical application effect of TIPS has been more and more affirmed [44–46].

Interventional therapy is an important treatment method for esophagogastric variceal bleeding in cirrhotic portal hypertension, and some surgical methods may vary according to the specific conditions of different patients. Interventional therapy is only part of the comprehensive treatment of esophageal variceal bleeding in cirrhotic portal hypertension. How to rationally use the existing treatment methods for these bleeding patients needs to be carefully analyzed clinically to maximize the benefits of the patients. Even in patients after TIPS, in addition to the necessary anticoagulation therapy, the treatment of the cause is also very important.

## 3. Combined Interventional and Laparoscopic Surgery

Interventional procedures can significantly reduce portal pressure by TIPS or reduction of splenic artery blood flow (PSE/SATE). Clinical studies [47, 48] have shown that early TIPS combined with endoscopic therapy can significantly improve the treatment success rate and prognosis of EVB patients. There are also studies [49] suggesting that early TIPS combined with endoscopic therapy can effectively prevent the recurrence of EVB, but it does not improve the survival rate. PSE combined with endoscopic techniques in the treatment of EVB can effectively control acute bleeding and prevent the occurrence of rebleeding [31]. It can also bring good clinical results in patients who cannot implement TIPS [50]. Taniai et al. [23] studied the recurrence rate of GOV at 6 months, 1 year, and 2 years after surgery in patients who received EVL and PSE combined therapy and patients who were treated with EVL alone or EIS alone, and the results showed that the recurrence rate was significantly higher in the combination therapy group than that in the EVL or EIS alone treatment group [24]. Murata et al. [25] conducted a one-year follow-up of patients after PSE, and the results showed that the cholinesterase, total cholesterol, total protein, albumin, and prothrombin time were all obtained within one year after PSE, continuous improvement. Compared with PSE, SATE also has the advantages of simple technique, can quickly reduce portal pressure, and has a significant effect on EVB control and prevention, and its complications are mild and easy to tolerate. It can be safely used in Child grade C patients [26, 51–54]. Although there is no clinical report of combining it with endoscopic therapy for EVB control and prevention, the combined therapy of splenic artery embolization and endoscopic therapy is theoretically capable of rapidly reducing portal pressure. It has the advantages of occluding submucosal varicose veins and can improve the liver function reserve status of patients to a certain extent and is not limited by Child classification. It is necessary to conduct further comparative studies on the clinical efficacy of this combination therapy.

## 4. Consensus Opinion

Limited by the lack of high-quality clinical studies, there is currently no widely accepted consensus on GV treatment strategies. For secondary prevention of GV, the American Academy of Liver Diseases recommends interventional therapy as first-line therapy and endoscopic therapy as second-line therapy [55]. British guidelines recommend endoscopic tissue glue injection as first-line therapy and TIPS as second-line therapy [56]. The most influential Baveno consensus in the world believes that endoscopic tissue glue injection, beta-blockers, endoscopic combined drug therapy, or interventional therapy (TIPSS) can be used to prevent GV rebleeding [57]. At the same time, both the Baveno consensus and the British guidelines believe that BRTO treatment of GV deserves further exploration.

## 5. Summary

In conclusion, endoscopy (EIS and EVL) and interventional therapy (PTVE, BRTO, and TIPS) are minimally invasive treatments that can effectively control and prevent GV bleeding. However, a large number of clinical studies are needed to distinguish the risks, pros, and cons of different surgical procedures and their application value in different populations. We should choose an individualized treatment strategy according to the specific situation of the patient: (1) whether it is used for the control of acute bleeding or mainly for the primary and secondary prevention of GV; (2) the diameter of the portal vein, whether there is portal vein thrombosis; (3) whether there is a large amount of ascites, shunt encephalopathy, hepatocellular carcinoma, etc.; (4) whether the patient's vital signs are stable and the liver function reserve; and (5) whether the hospital has technical means and equipment. All of these factors may influence the choice of treatment regimen. Both gastroscopy and interventional therapy have their own advantages and disadvantages. The ideal strategy is the combined application of gastroscopy and interventional therapy to learn from each other's strengths. We look forward to exploring the best treatment strategies for GV patients through rigorously designed prospective clinical studies.

## Data Availability

The data presented in the study may be made available from the corresponding author upon reasonable request.
